# Predictive Factors of Cardiac Mortality Following TEER in Patients with Secondary Mitral Regurgitation

**DOI:** 10.3390/jcm13030851

**Published:** 2024-02-01

**Authors:** Teruhiko Imamura, Shuhei Tanaka, Ryuichi Ushijima, Nobuyuki Fukuda, Hiroshi Ueno, Koichiro Kinugawa, Shunsuke Kubo, Masanori Yamamoto, Mike Saji, Masahiko Asami, Yusuke Enta, Masaki Nakashima, Shinichi Shirai, Masaki Izumo, Shingo Mizuno, Yusuke Watanabe, Makoto Amaki, Kazuhisa Kodama, Junichi Yamaguchi, Yoshifumi Nakajima, Toru Naganuma, Hiroki Bota, Yohei Ohno, Masahiro Yamawaki, Kazuki Mizutani, Toshiaki Otsuka, Kentaro Hayashida, on behalf of the OCEAN-Mitral Investigators

**Affiliations:** 1 Second Department of Internal Medicine, University of Toyama, Toyama 930-0194, Japanhueno@med.u-toyama.ac.jp (H.U.);; 2 Department of Cardiology, Kurashiki Central Hospital, Kurashiki 710-0052, Japan; sk12750@kchnet.or.jp; 3 Department of Cardiology, Toyohashi Heart Center, Toyohashi 441-8071, Japan; 4 Department of Cardiology, Nagoya Heart Center, Nagoya 461-0045, Japan; 5 Department of Cardiology, Gifu Heart Center, Gifu 500-8384, Japan; 6 Department of Cardiology, Sakakibara Heart Institute, Tokyo 183-0003, Japan; 7 Division of Cardiovascular Medicine, Department of Internal Medicine, Faculty of Medicine, Toho University, Tokyo 143-8540, Japan; 8 Division of Cardiology, Mitsui Memorial Hospital, Tokyo 101-8643, Japan; 9 Department of Cardiology, Sendai Kosei Hospital, Sendai 980-0873, Japannakashima.ma.71@gmail.com (M.N.); 10 Division of Cardiology, Kokura Memorial Hospital, Kitakyushu 802-8555, Japan; 11 Division of Cardiology, St. Marianna University School of Medicine Hospital, Kawasaki 216-8511, Japan; 12 Department of Cardiology, Shonan Kamakura General Hospital, Kanagawa 247-8533, Japan; shingo.waterfield@gmail.com; 13 Department of Cardiology, School of Medicine, Teikyo University, Tokyo 173-8606, Japan; 14 Department of Cardiology, National Cerebral and Cardiovascular Center, Suita 564-8565, Japan; amaki@ncvc.go.jp; 15 Division of Cardiology, Saiseikai Kumamoto Hospital Cardiovascular Center, Kumamoto 861-4193, Japan; 16 Department of Cardiology, Tokyo Woman’s Medical University, Tokyo 162-8666, Japan; 17 Division of Cardiology, Department of Internal Medicine, Iwate Medical University, Iwate 028-3694, Japan; 18 Department of Cardiology, New Tokyo Hospital, Chiba 270-2232, Japan; 19 Department of Cardiology, Sapporo Higashi Tokushukai Hospital, Sapporo 065-0033, Japan; 20 Department of Cardiology, School of Medicine, Tokai University, Isehara 259-1193, Japan; 21 Department of Cardiology, Saiseikai Yokohama City Eastern Hospital, Kanagawa 230-0012, Japan; 22 Division of Cardiology, Department of Medicine, Faculty of Medicine, Kinki University, Osaka 577-8502, Japan; 23 Department of Hygiene and Public Health, Nippon Medical School, Tokyo 113-8602, Japan; 24 Department of Cardiology, School of Medicine, Keio University, Tokyo 160-8582, Japan

**Keywords:** valvular disease, heart failure, hemodynamics, inotropes, MitraClip

## Abstract

**Background:** Transcatheter edge-to-edge mitral valve repair (TEER) has emerged as a viable approach to addressing substantial secondary mitral regurgitation. In the contemporary landscape where ultimate heart failure-specific therapies, such as cardiac replacement modalities, are available, prognosticating a high-risk cohort susceptible to early cardiac mortality post-TEER is pivotal for formulating an effective therapeutic regimen. **Methods:** Our study encompassed individuals with secondary mitral regurgitation and chronic heart failure enlisted in the multi-center (Optimized CathEter vAlvular iNtervention (OCEAN)-Mitral registry. We conducted an assessment of baseline variables associated with cardiac death within one year following TEER. **Results:** Amongst the 1517 patients (median age: 78 years, 899 males), 101 experienced cardiac mortality during the 1-year observation period after undergoing TEER. Notably, a history of heart failure-related admissions within the preceding year, utilization of intravenous inotropes, and elevated plasma B-type natriuretic peptide levels emerged as independent prognosticators for the primary outcome (*p* < 0.05 for all). Subsequently, we devised a novel risk-scoring system encompassing these variables, which significantly stratified the cumulative incidence of the 1-year primary outcome (16%, 8%, and 4%, *p* < 0.001). **Conclusions**: Our study culminated in the development of a new risk-scoring system aimed at predicting 1-year cardiac mortality post-TEER.

## 1. Introduction

Secondary mitral regurgitation (MR) is associated with mortality and morbidity in patients with chronic heart failure [1]. Transcatheter edge-to-edge mitral valve repair (TEER) using the MitraClip system (Abbott Vascular, Menlo Park, CA, USA) has been introduced with favorable post-procedural clinical outcomes when appropriately performed in carefully selected patients [2]. TEER is especially effective in preventing cardiac death, rather than comorbidity-related adverse events, considering its therapeutic target. The MitraClip system obtained Food and Drug Administration approval for the primary MR in 2013 and the secondary MR in 2019 given accumulating evidence [3,4,5,6]. This system has also been available in Japan since 2018, based on the favorable outcomes of the feasibility trial [7].

Aggressive titration of guideline-directed medical therapy (GDMT) is key to improving clinical outcomes and preventing cardiac death in patients with chronic heart failure and secondary MR [8,9]. GDMT can be strengthened even after TEER, which stabilizes hemodynamics and allows for further up-titration of medications. Cardiac replacement therapy, including durable ventricular assist device implantation (LVAD) and heart transplantation, is another key to ameliorating secondary MR by hemodynamic stabilization and cardiac reverse remodeling via mechanical unloading without direct intervention to the impaired mitral valve [10].

Some patients may be at a high risk of immediate cardiac death, including dependence on cardiac replacement therapy, even after a successful TEER. Such patients are good candidates for these other intensive cardiac therapies, with or without TEER [11]. For example, patients with a high risk of cardiac death may skip TEER and receive cardiac replacement therapy, if indicated. Others with a high risk of cardiac death may receive aggressive medical therapy immediately after TEER together with optimal shared decision-making. Thus, it is of great importance for clinicians to clarify such a high-risk cohort before TEER. 

In this investigation, we examined the baseline characteristics linked to premature cardiac mortality following TEER by utilizing extensive multi-center registry data. Moreover, our aim was to devise a risk-scoring system that forecasts the likelihood of early cardiac mortality post-TEER. 

## 2. Methods

### 2.1. Participant Selection

This retrospective study utilized data from the Optimized CathEter vAlvular iNtervention (OCEAN)-Mitral registry, a prospective, investigator-initiated, multi-center endeavor aimed at evaluating the safety and effectiveness of TEER in patients with significant MR unresponsive to optimal medical therapy. The registry comprises comprehensive information from 21 well-established large-scale Japanese institutions and covers an ongoing period. Data between April 2018 and June 2021 were retrospectively analyzed for this specific investigation. The registry is independent of industrial influence. 

Participants were categorized into two groups based on MR etiology: primary MR and secondary MR. Patients diagnosed with primary MR were excluded from the study cohort. Consequently, only individuals with secondary MR constituted the included cohort.

This study received registration in the University Hospital Medical Information Network Clinical Trials Registry (UMIN000023653). The research protocol obtained approval from the Institutional Review Board at each participating hospital, adhering to the principles outlined in the Declaration of Helsinki.

### 2.2. TEER Procedure

The decision to proceed with TEER was made by a multidisciplinary local heart team comprising interventional cardiologists, cardiothoracic surgeons, imaging cardiologists, medical engineers, and other skilled healthcare professionals. The severity of MR was quantified using the proximal isovelocity surface area method by the board-certified echocardiography experts at each institute. The patients, by principle, fulfilled the COAPT criteria and were unsuitable for mitral valve surgery. This decision-making process involved thorough discussions with the patients and their families, ensuring dedicated procedures were followed with informed consent [12].

TEER utilizing the MitraClip system adheres to standardized protocols [2]. The procedure was performed under general anesthesia under fluoroscopic and transesophageal echocardiographic guidance [13]. Commencing with a transseptal puncture through femoral vein access, a 24-Fr guide catheter was advanced into the left atrium. Subsequently, the clip delivery system was positioned above the origin of the MR jet and further advanced into the left ventricular cavity. The mitral leaflets were then engaged, and the clip was closed to bring the leaflets into approximation. If satisfactory reduction of the MR was attained, the clip was released. In cases requiring additional MR reduction, consideration was given to a potential second clipping based on residual MR assessment and the mean pressure gradient across the mitral valve [14].

### 2.3. Clinical Variables Obtained

The clinical variables gathered encompassed baseline characteristics pre-TEER, encompassing demographic information, comorbidities, laboratory findings, echocardiographic evaluations, and medication records. Specifically, the count of heart failure admissions within the preceding year was acquired. Medication data encompassed the administration of intravenous inotropes. Echocardiographic assessments adhered to the guidelines set forth by the American Society of Echocardiography [15].

Post-procedural outcomes encompassed echocardiographic evaluations related to valvular disease, the duration of hospitalization, in-hospital mortality, and the collection of adverse event data. The observation period lasted for one year following the TEER procedure. The primary outcome was explicitly categorized as cardiac mortality, inclusive of deaths attributed to cardiac conditions and instances necessitating cardiac replacement therapy (such as LVAD implantation and heart transplantation). 

### 2.4. Statistical Analyses

Continuous variables were depicted as medians along with their interquartile ranges, while categorical variables were represented by numbers and corresponding percentages. Baseline characteristics with potential clinical relevance were subjected to time-to-event analysis to forecast the primary outcome, utilizing Cox proportional hazard ratio regression analysis. Variables displaying significance in univariable analyses with a threshold of *p* < 0.05 were incorporated into the subsequent multivariable analyses, employing a stepwise method. Subsequently, a robust multivariable model was formulated, comprising only significant variables with a *p*-value < 0.05.

To develop a novel risk score, the hazard ratio of each variable was weighted accordingly. Time-dependent receiver operating characteristics analysis with the Youden method was conducted to determine cutoffs for this score in predicting the primary outcome. The patient cohort was stratified into three distinct risk categories: low-risk, intermediate-risk, and high-risk groups based on this score. Subsequently, post-procedural outcomes were compared among these groups using Fischer’s exact test.

A sub-analysis was exclusively performed within the high-risk group to further elucidate an exceptionally high-risk subset. Cox proportional hazard ratio regression analysis was reiterated for the primary outcome among potential post-procedural variables specifically within this cohort.

All statistical computations were executed using SPSS Statistics 23.0 software (IBM Corp, Armonk, NY, USA). A significance threshold of two-sided *p*-values < 0.05 was employed for all analyses.

## 3. Results

### 3.1. Baseline Characteristics

Out of the 2150 patients who underwent TEER and were registered, 633 individuals with primary MR were excluded from the study, resulting in the final inclusion of 1511 patients (Table 1).

The median age was 78 (71, 84) years, and 899 were male patients. All patients had heart failure with secondary MR. The New York Heart Association functional class was III or IV in 982 (65%) patients. A median heart failure admission time within the past year was 1 (1, 2). In detail, 376 (25%) patients had no history of heart failure admission, and 670 (44%) had one heart failure admission. Approximately 10% had heart failure admission equal to or above 3 times previously. A total of 1135 patients (75%) had at least one heart failure admission within the past year. Intravenous inotropes were administered in 176 (12%) patients. 

Echocardiographic parameters demonstrated a median left ventricular end-diastolic diameter of 60 (53, 67) mm and a median left ventricular ejection fraction of 36% (29%, 48%). All patients exhibited moderate or severe MR, with a median effective regurgitant orifice area of 30 (22, 40) cm^2^ and a median regurgitant volume of 48 (36, 61) mL.

The patients received guideline-directed medical therapy tailored for chronic heart failure, with a median equivalent dose of furosemide recorded at 20 (20, 40) mg per day.

### 3.2. One-Year Primary Outcome

Throughout the 1-year observational period post-procedure, 101 patients experienced cardiac mortality, constituting the primary outcome of this investigation. Within this outcome, 100 deaths were attributable to cardiac causes, while one patient underwent ventricular assist device implantation. 

Thirteen pre-specified potential risk factors were considered for the time-to-event analysis. Following the analysis, it was revealed that the number of heart failure admissions within the preceding year, the utilization of intravenous inotropes, and the common logarithm of plasma B-type natriuretic peptide were independently associated with the primary outcome (*p* < 0.05 for all; Table 2).

### 3.3. New Risk Score for the Primary Outcome

Utilizing the hazard ratio of each parameter, a risk score was formulated to predict 1-year cardiac mortality, calculated as follows: 1.28 × (number of heart failure admissions) + 1.95 × (utilization of intravenous inotropes) + 2.40 × (common logarithm of plasma BNP [pg/mL]). Subsequently, risk scores were computed for all participants, yielding a median score of 8.0 (6.7, 9.4) points.

**Figure 1:**
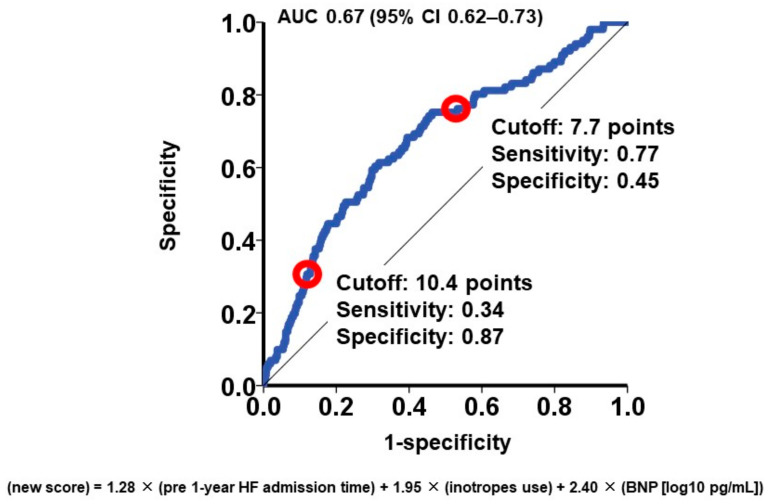
Receiver operating characteristics analysis for the impact of new score on the primary outcome. The primary outcome was cardiac death during the 1-year observation period after the MitraClip procedure. AUC, area under the curve; CI, confidence interval. Red circles represent cutoffs.

**Figure 2:**
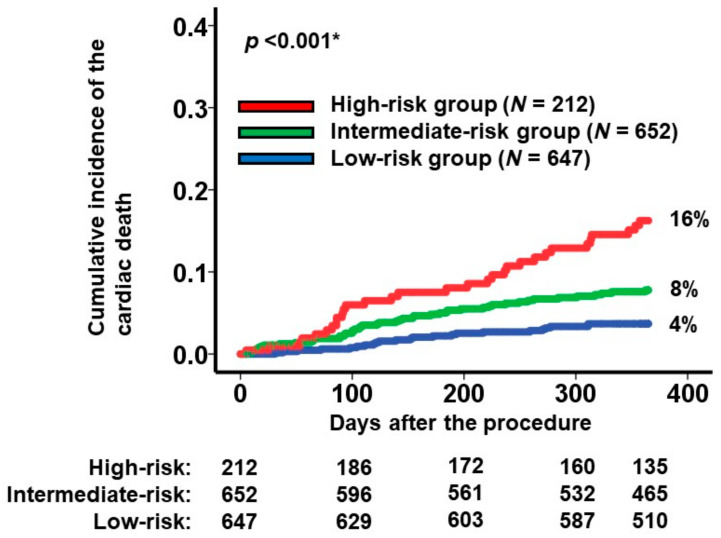
Cumulative incidence of the primary outcome during a 1-year observation period among all the participants. Patients were stratified into 3 groups according to the new scores: high-risk, intermediate-risk, and low-risk groups. The primary outcome was cardiac death. * *p* < 0.05 by log-rank test.

To categorize the patient cohorts into distinct risk groups, two cutoffs were established through receiver operating characteristics analysis for the primary outcome (area under the curve: 0.67 (95% confidence interval: 0.62–0.73); Figure 1). Accordingly, individuals were classified into the following risk categories: the low-risk group (scores < 7.7; *N* = 647), the intermediate-risk group (scores ranging between 7.7 and 10.4; *N* = 652), and the high-risk group (scores >10.4; *N* = 212). Notably, the cumulative incidences of the primary outcome displayed significant stratification among these three risk groups (16%, 8%, and 4% for the high-risk, intermediate-risk, and low-risk groups, respectively; *p* < 0.001; Figure 2).

### 3.4. Post-Procedural Complications

Following TEER, 222 patients (15%) exhibited persistent moderate or severe MR (Table 3). The median duration of the initial hospitalization post-procedure was 16 (9, 30) days. Thirty-two patients (2%) succumbed during this index hospitalization period. No occurrences of clip embolization were reported. Subsequent to discharge, 93 patients (6%) experienced a recurrence of moderate or greater MR, and a total of 319 patients (21%) faced readmissions due to heart failure.

Stratification of patients into three distinct risk-score-based groups (as elucidated below in Table 3) revealed notable differences in several outcomes. The high-risk group exhibited prolonged in-hospital stays, a heightened incidence of in-hospital mortality, and a greater occurrence of heart failure readmissions compared to both the low-risk and intermediate-risk groups (*p* < 0.05 for all comparisons).

### 3.5. Sub-Analysis to Further Risk Stratify the High-Risk Group

Within the high-risk cohort, comprising 212 patients identified using pre-procedure variables, four potential post-TEER variables, instead of pre-TEER variables, were scrutinized for their impact on the primary outcome (Table 4). Notably, an extended duration of hospitalization exhibited a significant association with the primary outcome, demonstrating a hazard ratio of 1.10 (per 10 days, 95% confidence interval: 1.01–1.21, *p* = 0.046). Patients who received intravenous inotropes necessitated longer hospital stays compared to those who did not receive intravenous inotropes: 30 (21, 48) days versus 15 (10, 26) days, *p* < 0.005.

**Figure 3:**
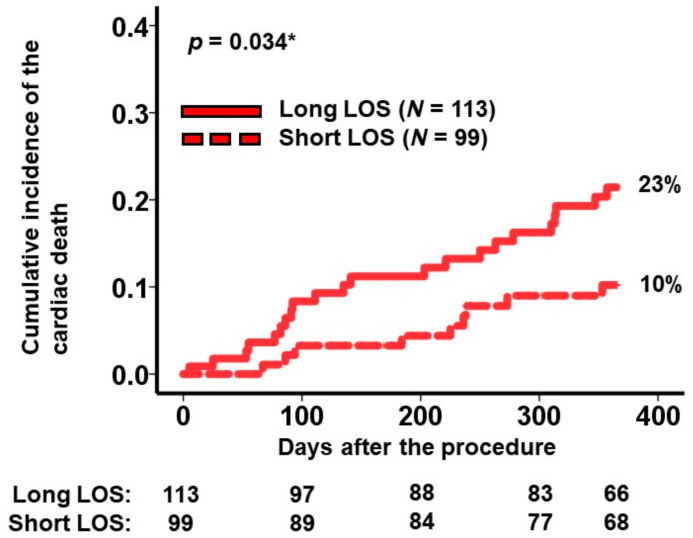
Cumulative incidence of the primary outcome during a 1-year observation period among the high-risk patients. Patients in the high-risk group were stratified into 2 groups according to the length of stay during index hospitalization. The primary outcome was cardiac death. LOS, length of stay. * *p* < 0.05 by log-rank test.

A cutoff for the length of stay predicting the primary outcome was calculated as 2.0 × 10 days by receiver operating characteristics analysis (area under the curve: 0.6 (95% confidence interval: 0.49–0.71, sensitivity: 0.71, specificity: 0.47)). Within the high-risk group, 113 patients experienced longer hospital stays exceeding 2.0 × 10 days. The cumulative incidence of the primary outcome in patients with prolonged hospitalizations (i.e., the very-high-risk cohort) was notably higher than in those with shorter hospital stays (23% versus 10%, *p* = 0.034; Figure 3).

## 4. Discussion

In this retrospective analysis utilizing prospectively collected data from the multi-center OCEAN-Mitral registry, our primary focus was on identifying risk factors associated with 1-year cardiac mortality and establishing a risk-scoring system based on these factors among patients with chronic heart failure and secondary MR undergoing TEER.

The majority of TEER procedures achieved successful outcomes, with over 80% of patients exhibiting mild or lesser residual MR and an in-hospital mortality rate of below 5%. Through our investigation, three specific risk factors were identified as significantly linked to 1-year cardiac mortality. These included a history of recurrent hospitalizations due to heart failure, utilization of intravenous inotropes, and elevated levels of plasma BNP. Subsequently, we developed a risk-scoring system that effectively stratified the cumulative incidence of 1-year cardiac mortality into three distinct risk categories.

Despite the efficacy of the risk score in predicting cardiac mortality, its application did not correlate with rates of residual or recurrent MR, nor did it significantly predict occurrences of post-procedural complications. It is noteworthy that among the patients categorized within the high-risk group, a post-TEER extended in-hospital stay was indicative of an even higher-risk subset (termed the “very high-risk cohort”). This observation highlights the potential for further risk stratification within the high-risk population based on the duration of hospitalization post-TEER.

### 4.1. Definition of the Primary Outcome

The primary outcome in this study was defined as cardiac death, encompassing fatalities attributed to heart-related causes and instances necessitating cardiac replacement therapy. It is notable that after undergoing TEER, some elderly patients with multiple comorbidities and advanced frailty may pass away due to non-cardiac diseases [16]. The emphasis of this study was specifically aimed at evaluating the influence of TEER in averting cardiac mortality rather than considering all-cause mortality. This focus allowed for a targeted examination of the impact of TEER on preventing cardiac death. Furthermore, our discussion revolved around exploring the possibility of ultimate cardiac-specific therapies for patients deemed to be at heightened risk of cardiac mortality post-TEER. This is the rationale for why we defined cardiac death as the primary outcome in this study, instead of all-cause mortality, which the previous literature has focused on [17,18].

### 4.2. Risk Factors Associated with Post-TEER Cardiac Death

The previous literature has also proposed several risk factors for the clinical outcomes after the MitraClip procedure. These include extra-cardiac parameters, such as the presence of chronic obstructive pulmonary disease and chronic kidney disease [17,19], probably because the primary endpoints of these studies were all-cause death and/or heart failure readmission, instead of cardiac death alone. 

Uniquely, we defined cardiac death as a primary endpoint in this study. This may be a reason identified risk factors in this study were all associated with heart: frequent heart failure hospitalizations (i.e., frequent flyer), intravenous inotropes use (dependence is unknown), and higher plasma BNP levels. Interestingly, these are well-known factors associated with INTERMACS profile 3 or 4. INTERMACS profile 3 is assigned to individuals dependent on inotropes. INTERMACS profile 4 is assigned to frequent flyers with repeated heart failure hospitalizations. Patients assigned to INTERMACS profile 3 or 4 are good candidates for durable LVAD implantation unless there are other contraindications [20,21]. Our risk score is superior to the conventional INTERMACS profile because the INTERMACS profile is relatively subjective. Of note, we cannot completely define the dependence on inotropes. 

Durable LVAD therapy alone can ameliorate most of the secondary MR in patients with advanced heart failure, without any concomitant interventions to the mitral valve [10]. Some of the patients assigned to the high-risk group may be good candidates for durable LVAD implantation, instead of TEER, whereas we should consider the risk of LVAD-related complications [22].

Anatomical features were not significantly associated with the primary outcome in this study, including the left ventricular size. This is probably because we strictly obeyed the indication of TEER by referencing the findings of COAPT and MITRA-FR trials [5,6]. TEER is not indicated in patients with a left ventricle that is too remodeled in the current era [23]. In addition to these anatomical indications, we emphasize that several clinical risk factors, which we found here, are of great importance to consider the indication of TEER. 

The rates of post-procedure residual/recurrent MR and procedure-related complications were not stratified by the risk score. These adverse events were well managed by a successful TEER, similar to the COAPT trial [5]. Thus, cardiac death may develop even after the strict management of significant MR in the high-risk cohorts. 

### 4.3. Clinical Implications of Our Findings

Considering the relatively acceptable outcomes, TEER may be recommended even in patients assigned to the high-risk group. Before TEER, careful shared decision-making is highly recommended for the patients and their relatives to inform them about the procedural risk.

Durable LVAD therapy may be recommended, instead of TEER, if they have a good indication for durable LVAD [24]. Durable LVAD can prevent cardiac death, along with ameliorating severe MR without concomitant intervention to the mitral valve via hemodynamic improvement and cardiac reverse remodeling. 

Others in the high-risk group should be carefully followed even after the successful TEER. A good control of MR without any procedure-related complications does not guarantee favorable outcomes in this cohort. 

Especially patients with longer in-hospital stays post-TEER have a very high risk of future cardiac death. They may have taken a longer time weaning from intravenous inotropes after TEER and adjusting to heart failure medications. Aggressive therapies to prevent cardiac death may be required in this cohort, including the optimization of heart failure medications, cardiac resynchronization therapy, and durable LVAD implantation, if indicated [25,26]. TEER may allow for hemodynamic stabilization and give further opportunities to strengthen these therapies that could not be tolerated before TEER. 

### 4.4. Limitations

The limitations of our study include our efforts to account for potential confounding variables; however, there remains a possibility of other unexplored factors associated with the primary outcome that were not considered in our analysis. The prognostic impact of invasively measured hemodynamics following TEER could be significant, but these measurements were not universally conducted across all participating institutions. The echocardiographic data, including the assessments of mitral regurgitation severity, were not assessed in an independent core laboratory. Of note, we lack comprehensive echocardiographic data regarding detailed right ventricular function, which may also have a prognostic impact after TEER. Instead, these evaluations were performed by experienced echocardiographic physicians certified by relevant boards. 

We included dependence on cardiac replacement therapy also in cardiac death, the primary endpoint in this study. In this study, we encountered only one patient who required durable LVAD implantation. The detailed association between our risk score and cardiac replacement therapy remains uncertain. Our score is simple and three items can be obtained easily without any unique examinations. Nevertheless, we may need a calculator for the score calculation. We used the Youden method to calculate the cutoffs. However, any other optimal cutoffs may better stratify the risk of the cohorts. 

Due to the relatively limited number of events and the challenge of constructing a robust multivariable model, we were unable to establish a validation cohort. Given the racial and geological discrepancies in the baseline characteristics, we should pay attention to simply applying our findings to other countries. Consequently, it is essential that our findings are corroborated by validation studies conducted on another larger-scale cohort to enhance the robustness and generalizability of our observations.

## 5. Conclusions

Our study leveraged extensive data from the multi-institute OCEAN-Mitral registry to discern risk factors associated with 1-year cardiac mortality subsequent to TEER in patients grappling with chronic heart failure and secondary MR. Notably, we identified several risk determinants, namely, recurrent heart failure admissions, the utilization of intravenous inotropes, and elevated plasma BNP levels.

The formulation of a novel risk-scoring system incorporating these identified risk factors effectively delineated distinct risk strata regarding the cumulative incidence of our primary outcome. This stratification signifies the potential for risk assessment and segmentation within this patient cohort.

## Figures and Tables

**Table 1:** Baseline characteristics.

	Total (*N* = 1511)	Cardiac Death (*N* = 101)	No Cardiac Death (*N* = 1410)	*p*-Value
Demographics				
Age, years	78 (71, 84)	80 (74, 85)	78 (71, 84)	0.096
Male sex	899 (60%)	63 (63%)	836 (59%)	0.31
Body mass index, kg/m^2^	21.2 (18.9, 23.5)	20.4 (18.1, 22.7)	21.3 (19.0, 23.5)	0.036 *
NYHA class III or IV	982 (65%)	82 (82%)	900 (64%)	<0.001 *
Heart failure admission times within the past one year	1 (1, 2)	1 (1, 2)	1 (0, 2)	0.014 *
0 time	376 (25%)	21 (21%)	355 (25%)	0.010 *
1 time	670 (44%)	35 (35%)	635 (45%)	0.010 *
2 times	305 (20%)	21 (21%)	284 (20%)	0.010 *
≥3 times	160 (11%)	24 (24%)	136 (10%)	0.010 *
Etiology of MR				
Primary MR	0 (0%)	0 (0%)	0 (0%)	-
Secondary MR	1511 (100%)	101 (100%)	1410 (100%)	-
Comorbidity				
Hypertension	996 (66%)	59 (59%)	937 (66%)	0.063
Dyslipidemia	862 (57%)	44 (44%)	818 (58%)	0.003 *
Atrial fibrillation	976 (65%)	73 (73%)	903 (64%)	0.057
History of ventricular tachyarrhythmia	199 (13%)	21 (21%)	178 (13%)	0.018 *
History of coronary intervention	593 (39%)	34 (34%)	549 (39%)	0.62
History of open heart surgery	177 (12%)	8 (8%)	169 (12%)	0.14
History of stroke	173 (11%)	14 (14%)	159 (11%)	0.26
PM/ICD/CRT-D/CRT-P	105/102/189/23	7/7/20/2	98/95/169/21	0.23
Laboratory data				
Hemoglobin, g/dL	11.6 (10.4, 12.9)	10.7 (9.7, 12.0)	11.7 (10.4, 13.0)	<0.001 *
Serum creatinine, mg/dL	1.3 (1.0, 1.9)	1.5 (1.1, 2.2)	1.3 (1.0, 1.9)	0.077
Plasma B-type natriuretic peptide, log_10_ pg/mL	2.7 (2.4, 2.9)	2.9 (2.6, 3.2)	2.6 (2.3, 2.9)	<0.001 *
Echocardiographic data				
Left ventricular end-diastolic diameter, mm	60 (53, 67)	62 (54, 70)	60 (53, 67)	0.046 *
Left ventricular ejection fraction, %	36 (29, 48)	33 (26, 44)	36 (29, 49)	0.004 *
<40%	907 (60%)	72 (72%)	835 (59%)	0.056
40–49%	260 (17%)	13 (13%)	247 (18%)	0.056
≥50%	344 (23%)	16 (16%)	328 (23%)	0.056
Left atrial volume index, mL/m^2^	76 (58, 104)	90 (67, 116)	76 (58, 104)	0.003 *
Effective regurgitant orifice area, cm^2^	30 (22, 40)	32 (25, 45)	30 (22, 40)	0.38
Regurgitant volume, mL	48 (36, 61)	48 (37, 61)	47 (36, 61)	0.67
Moderate or greater tricuspid regurgitation	524 (35%)	39 (39%)	485 (34%)	0.14
Medications				
Beta-blocker	1238 (82%)	79 (79%)	1159 (82%)	0.19
Renin-angiotensin system inhibitor	941 (62%)	61 (61%)	880 (62%)	0.38
Mineralocorticoid receptor antagonist	876 (58%)	64 (64%)	812 (58%)	0.15
Sodium-glucose cotransporter 2 inhibitor	192 (13%)	18 (18%)	174 (12%)	0.079
Dose of furosemide, mg/day	20 (20, 40)	20 (20, 40)	20 (20, 40)	0.84
Intravenous inotropes	176 (12%)	23 (23%)	153 (11%)	0.001 *

NYHA, New York Heart Association; MR, mitral regurgitation. PM, pacemaker; ICD, implantable cardioverter-defibrillator; CRT-D, cardiac resynchronization therapy with defibrillator; CRT-P, cardiac resynchronization therapy with pacemaker. Continuous variables are stated as median and interquartile and compared between the two groups using the Mann–Whitney U test. Categorical variables are stated as numbers and percentages and compared between the two groups using the Chi-square test or Fischer’s exact test. * *p* < 0.05.

**Table 2:** Potential variables associated with future cardiac death.

	Univariable Analysis		Multivariable Analysis		Multivariable Analysis	
	Hazard Ratio (95% CI)	*p*-Value	Hazard Ratio (95% CI)	*p*-Value	Hazard Ratio (95% CI)	*p*-Value
Age, years	1.02 (0.99–1.04)	0.14				
Male sex	1.14 (0.76–1.71)	0.52				
Body mass index, kg/m^2^	0.95 (0.89–1.01)	0.056				
HF admission number within the past one year	1.33 (1.14–1.54)	<0.001 *	1.26 (1.07–1.47)	0.005 *	1.28 (1.09–1.50)	0.002 *
Atrial fibrillation	1.46 (0.95–2.26)	0.087				
History of ventricular tachyarrhythmia	1.74 (1.08–2.81)	0.024 *	1.46 (0.87–2.42)	0.15		
Intravenous inotropes use	2.50 (1.57–3.97)	<0.001 *	1.81 (1.11–2.94)	0.017 *	1.95 (1.21–3.13)	0.006 *
Serum creatinine, mg/dL	1.07 (0.96–1.20)	0.20				
Plasma BNP, log_10_ pg/mL	2.53 (1.71–3.75)	<0.001 *	2.39 (1.55–3.67)	<0.001 *	2.40 (1.59–3.63)	<0.001 *
ECG width, msec	1.01 (0.99–1.02)	0.19				
LVDD, mm	1.02 (1.01–1.04)	0.035 *	1.01 (0.098–1.03)	0.70		
LVEF, %	0.98 (0.97–0.99)	0.012 *	0.99 (0.98–1.02)	0.82		
Furosemide dose, mg/day	1.01 (0.99–1.02)	0.21				

CI, confidence interval; HF, heart failure; BNP, B-type natriuretic peptide; LVDD, left ventricular ejection fraction; LVEF, left ventricular ejection fraction. Cox proportional hazard ratio regression analysis was performed. Variables with *p* < 0.05 in the univariable analyses were included in the multivariable analysis. * *p* < 0.05.

**Table 3:** Post-procedural outcome stratified by the new risk score.

	Low-Risk (*N* = 647)	Intermediate-Risk (*N* = 652)	High-Risk (*N* = 212)	*p*-Value
Post-procedure moderate or greater MR	94 (15%)	93 (14%)	35 (17%)	0.72
Post-procedure moderate or greater TR	195 (30%)	160 (25%)	60 (28%)	0.074
Index hospitalization days	11 (7, 20)	17 (10, 31)	21 (12, 36)	<0.001 *
In-hospital death	1 (1%)	25 (4%)	6 (3%)	<0.001 *
Access site-related complication	13 (2%)	17 (3%)	3 (1%)	0.54
Acute kidney injury	10 (2%)	23 (3%)	5 (3%)	0.073
Single leaflet device attachment	10 (2%)	8 (1%)	2 (1%)	0.77
Leaflet tear	6 (1%)	9 (1%)	6 (3%)	0.12
Clip embolization	0 (0%)	0 (0%)	0 (0%)	-
Recurrence of moderate or greater MR	47 (7%)	37 (6%)	9 (4%)	0.24
Redo clipping	2 (1%)	0 (0%)	0 (0%)	0.31
Heart failure readmission	100 (15%)	150 (23%)	69 (33%)	<0.001 *

MR, mitral regurgitation; TR, tricuspid regurgitation. Continuous variables are stated as median and interquartile and compared between the groups using the Wilcoxon signed-rank test. Categorical variables are stated as numbers and percentages and compared between the groups using Fischer’s exact test. * *p* < 0.05.

**Table 4:** Post-procedure factors associated with the primary outcome among the high-risk cohort.

	Hazard Ratio (95% CI)	*p*-Value
Post-procedure moderate or greater MR	0.97 (0.37–2.52)	0.95
Post-procedure moderate or greater TR	1.57 (0.75–3.27)	0.23
Length of hospital stay, ×10 days	1.10 (1.01–1.21)	0.046 *
Acute kidney injury	1.45 (0.20–10.6)	0.72

CI, confidence interval; MR, mitral regurgitation; TR, tricuspid regurgitation. Potential variables obtained after the procedure were included in the univariable Cox proportional hazard ratio regression analyses for the primary outcome. * *p* < 0.05.

## Data Availability

Data are available upon reasonable request from the corresponding author.
